# GLUD1 Inhibition Disrupts Glutamate Homeostasis and Induces Metabolic and Redox Stress in Gliomas

**DOI:** 10.3390/cells15151401

**Published:** 2026-08-03

**Authors:** Malgorzata Trybula, Małgorzata Łysiak, Emilia Wiechec, Annika Malmström, Peter Söderkvist

**Affiliations:** 1 Department of Biomedical and Clinical Sciences, Division of Cell and Neurobiology, Linköping University, 581 83 Linköping, Sweden; emilia.wiechec@liu.se (E.W.); annika.malmstrom@regionostergotland.se (A.M.); peter.soderkvist@liu.se (P.S.); 2 Clinical Genomics Linköping and Department of Biomedical and Clinical Sciences, Linköping University, 581 83 Linköping, Sweden; malgorzata.lysiak@liu.se; 3 Department of Otorhinolaryngology in Linköping, Anesthetics, Operations and Specialty Surgery Center, 581 85 Linköping, Sweden; 4 Faculty of Medicine, Academy of Silesia, Rolna 43, 40-555 Katowice, Poland; 5 Clinical Department of Geriatrics and Palliative Medicine, 581 85 Linköping, Sweden; 6 Science for Life Laboratory, Linköping University, 581 85 Linköping, Sweden

**Keywords:** glioma, cancer metabolism, GLUD1, glutamate dysregulation, redox

## Abstract

Glutamate dehydrogenase (GLUD1) links glutamine metabolism and redox regulation, yet its prognostic and functional relevance across different glioma subtypes warrants further study. Here, we show that *GLUD1* expression was inversely associated with tumor grade and positively associated with survival across glioma subtypes, a relationship not fully recapitulated by broader glutaminolysis-related gene signatures. To investigate the consequences of GLUD1 inhibition, we treated endogenous *IDH*-mutant and *IDH*-wildtype glioma cell lines with the reported GLUD1 inhibitor R162. GLUD1 inhibition reduced viability in all cell lines tested. This effect was not rescued by α-ketoglutarate (α-KG) supplementation, indicating that impaired tricarboxylic acid (TCA) cycle anaplerosis was not the primary mechanism underlying GLUD1 dependency. Instead, GLUD1 inhibition caused intracellular glutamate accumulation, increased reactive oxygen species (ROS), γ-H2AX induction, and elevated intracellular calcium, while complementary in silico analyses predicted disruption of mitochondrial membrane potential following R162 exposure. Together, these findings indicate that GLUD1 inhibition induces metabolic and redox stress associated with disrupted glutamate and calcium homeostasis and DNA damage. Our findings distinguish the favorable prognostic value of *GLUD1* expression from the cellular vulnerability revealed by its inhibition, supporting further investigations of GLUD1 as both a prognostic biomarker and potential therapeutic target in glioma.

## 1. Introduction

**Figure 1:**
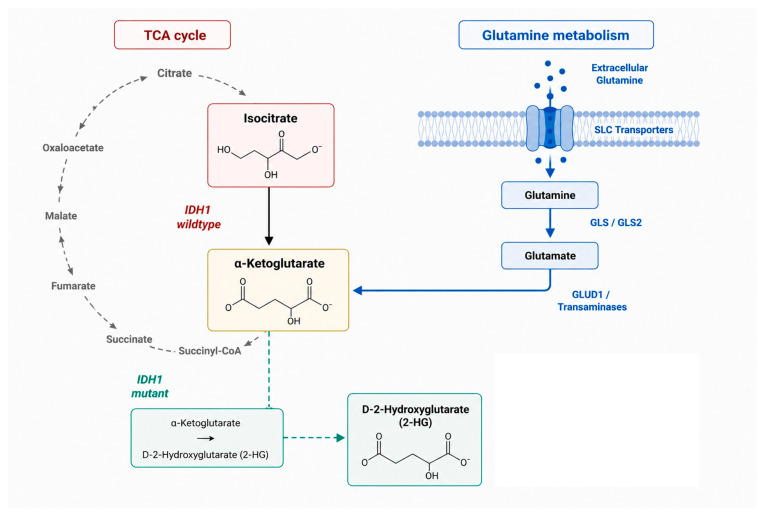
Glutamine metabolism pathway in IDH-wildtype and IDH-mutant gliomas. Glutamine is transported into cells via solute carrier (SLC) transporters, including SLC1A5 and SLC1A7, and is converted to glutamate by glutaminase (GLS). Glutamate is subsequently metabolized to α-ketoglutarate by glutamate dehydrogenase (GLUD1) or transaminases. α-Ketoglutarate represents an important metabolic intersection between glutamine metabolism and the tricarboxylic acid (TCA) cycle. In IDH1-wildtype cells, α-ketoglutarate can support TCA cycle anaplerosis, whereas mutant IDH1 converts α-ketoglutarate to the oncometabolite D-2-hydroxyglutarate. These distinct metabolic fates of α-ketoglutarate may influence anaplerosis and contribute to the different metabolic environments of IDH1-wildtype and IDH1-mutant gliomas.

Gliomas are among the most aggressive brain tumors, with current therapies mainly focusing on prolonging survival [1]. Recent research has revealed that glioma growth is tightly linked to metabolic reprogramming, particularly glutamine metabolism, also known as glutaminolysis [2]. In this pathway, glutamine is converted to glutamate by glutaminase (GLS), and glutamate is further oxidized to α-KG by GLUD1, feeding the TCA cycle to sustain energy production and biosynthesis (Figure 1) [3,4].

Among the molecular alterations that shape glioma metabolism, mutations in isocitrate dehydrogenase (*IDH1/2*) are particularly important. These mutations are recognized as tumor-initiating events in gliomas and reprogram cellular metabolism by promoting production of the oncometabolite 2-hydroxyglutarate (2-HG) instead of α-KG [5,6]. As a result, *IDH*-mutant tumors—such as oligodendrogliomas and astrocytomas—exhibit distinct metabolic dependencies compared to *IDH*-wildtype glioblastomas (GBMs). These metabolic vulnerabilities can be therapeutically exploited; for example, recent clinical data show that inhibition of mutant *IDH* prolongs progression-free survival in *IDH*-mutant glioma patients [7].

Since several cancers, including gliomas, depend on reprogrammed glutamine metabolism, key enzymes in this pathway—such as GLUD1—have been investigated for their functional significance in tumor development and progression [8,9,10,11]. GLUD1 is critical for glutamine metabolism and energy production, yet its role appears to vary across tumor types. In renal cell carcinoma, GLUD1 has been reported to act as a tumor suppressor [8], whereas in lung cancer it supports tumor progression [12]. In gliomas, recent studies have linked *GLUD1* expression to patient survival in *IDH*-mutant gliomas [13] and reported associations between *GLUD1* expression and genes involved in glutathione (GSH) metabolism [10,14]. Zhang et al. further demonstrated that GLUD1 regulates bioenergetics and redox homeostasis in glioma and that its inhibition suppresses GBM cell growth [15]. However, the prognostic significance of *GLUD1* across different glioma subtypes and the cellular consequences of its inhibition in these contexts remain incompletely understood. GLUD1 regulates glutamate metabolism, and glutamate occupies a central position at the intersection of cellular metabolism and redox regulation: it contributes to GSH synthesis but may also induce cellular stress when accumulated [16]. Thus, altered GLUD1 activity could affect glioma cell homeostasis by changing the balance between glutamate utilization and accumulation. We hypothesized that pharmacological GLUD1 inhibition would disrupt glutamate homeostasis and induce metabolic and oxidative stress, potentially enhancing the effects of radiation. To investigate this, we examined the prognostic relevance of *GLUD1* expression and investigated the cellular consequences of pharmacological GLUD1 inhibition using the reported GLUD1 inhibitor R162 in *IDH*-wildtype and *IDH*-mutant endogenous glioma models.

## 2. Materials and Methods

### 2.1. Cell Lines

The human GBM cell lines U3101 and U3123 were obtained from the Human Glioma Cell Culture (HGCC) biobank, Uppsala University, Sweden, and cultured as a monolayer in a 1:1 mixture of DMEM/F-12 (#11330032) and Neurobasal (#21103049) medium, supplemented with 1% B-27 (#125870101), 0.5% N-2 (#17502048) supplements, 100 U/mL of penicillin-streptomycin (#15140148; all from Thermo Fisher Scientific, Waltham, MA, USA), 20 ng/mL of Epidermal Growth Factor (EGF; #AF10015) and 10 ng/mL of recombinant human fibroblast growth factor (FGF-basic; #10018B; both from Thermo Fisher Scientific). The BT54 cell line, derived from an oligodendroglioma grade 3, was kindly provided by Professor Samuel Weiss (University of Calgary, Calgary, AB, Canada) and maintained as a suspension culture in NeuroCult Basal Medium supplemented with NeuroCult Supplement (#05751; both from Stem Cell Technologies, Vancouver, BC, Canada), 100 U/mL of penicillin-streptomycin, 20 ng/mL of EGF, 20 ng/mL of FGF and 2 μg/mL of heparin sulphate (#07980; Stem Cell Technologies). The molecular characteristics of these cell lines, including their *IDH* status and other relevant genomic alterations, have been previously characterized [17,18]. All cell lines were incubated at 37 °C in a humidified atmosphere with 5% CO_2_.

### 2.2. Study Subjects

Normalized RNA-seq data and clinical characteristics from the TCGA GBMLGG dataset were obtained from the Gliovis webpage (retrieved 24 March 2022, from: https://gliovis.bioinfo.cnio.es/). The TCGA cohort included 217 GBM, 235 astrocytoma and 154 oligodendroglioma samples. Tumors were classified according to the 2021 World Health Organization (WHO) classification of central nervous system tumors [19]. The expression data was used to perform unsupervised hierarchical clustering based on the varying expression of GRGs (*GLUD1*, *GOT1/2*, *GLS*, *GLS2*, *SLC1A5*, *SLC1A7*, *GLUL,* and *GPT1/2*) across all glioma samples. Clustering analysis was conducted using the pheatmap and cluster packages in RStudio.

### 2.3. GLUD1 Enzyme Activity

GLUD1 enzymatic activity was measured using GDH enzyme activity assay kit (#ab102527; Abcam, Cambridge, UK) according to the manufacturer’s instructions. Briefly, glioma cells were seeded in ultra-low attachment 96-well round-bottom plates (#7007; Corning, Corning, NY, USA) and incubated for 3 days at 37 °C in a humidified atmosphere with 5% CO_2_. On day three, 50% of the conditioned medium was removed, and the cells were treated with either 20 µM of R162 (#64302870; MedChemExpress, Monmouth Junction, NJ, USA) or 1% DMSO (#D8418; Merck, Darmstadt, Germany) as a vehicle control. The treatments were incubated for 1 h, after which cells were harvested and lysed using the assay buffer provided by the manufacturer. GLUD1 activity was measured on Spark 10 microplate reader (Tecan, Zurich, Switzerland). For measurements assessing the effect of calcium, 10 µg of total cell lysate was incubated with varying concentrations of CaCl_2_ (#1.02382; Merck) and EDTA (AC09651000; Scharlau Chemie, Barcelona, Spain), in the presence or absence of R162, for 10 min at room temperature (RT) prior to enzymatic activity measurement.

### 2.4. Viability

A total of 7500 glioma cells per well were seeded in ultra-low attachment 96-well round-bottom plates and incubated for 3 days at 37 °C in a humidified atmosphere with 5% CO_2_. On day three, 50% of the conditioned medium was removed, and the cells were treated with 20 μM of R162, either alone or in combination with radiation (9 Gy, single dose), DM-αKG (#13192046), fumarate (#624497; both from MedChemExpress) or NAC (Merck), dissolved in cell culture medium. The cells were incubated for an additional five days. Following treatment, CellTiter-Glo 3.0 reagent (#G9681; Promega, Madison, WI, USA) was added, and plates were shaken at 500 rpm for 5 min, followed by incubation for 30 min at RT. Luminescence was measured using Spark 10 plate reader (Tecan).

### 2.5. Measurement of Intracellular Glutamate Levels

Intracellular glutamate levels were measured using the Glutamate Assay Kit (#MAK330; Merck), following the manufacturer’s instructions. Briefly, after 24 h of incubation with the R162 inhibitor (20 μM), cells were harvested and washed with ice-cold PBS. The cell pellets were then resuspended in Assay Buffer and incubated on ice for 30 min with occasional vortexing. This was followed by centrifugation at 13,000× *g* for 10 min at 4 °C. The resulting supernatant was transferred to a 10K MWCO filter (#UFC5010; Millipore, Burlington, MA, USA) and centrifuged at 14,000× *g* for 15 min at 4 °C. After centrifugation, the filter was discarded, and 20 μL of flow-through was mixed with the reaction mix. Absorbance was then measured on Spark 10 plate reader at 565 nm over a period of 2 h, with readings taken every 30 min.

### 2.6. Western Blot

On day five of treatment, cells were harvested, washed twice with ice-cold PBS, and lysed in ice-cold RIPA buffer (#R0278) containing complete mini EDTA-free protease inhibitors (#11836170001; both from Merck). The lysates were incubated on ice for 30 min with occasional vortexing, followed by brief sonication. The lysates were then centrifuged at 13,000× *g* for 10 min, and protein concentration was determined using the BCA assay kit (#A55864; Thermo Fisher Scientific). Ten µg of total protein was mixed with 2× sample buffer (#1610737; BioRad, Hercules, CA, USA) supplemented with beta-mercaptoethanol (#M6250; Merck), denatured at 70 °C for 5 min, and loaded onto Mini-PROTEAN TGX 4–15% gels (#4568086; Bio-Rad). The separated proteins were transferred onto a PVDF membrane (#1704156; Trans-Blot Turbo PVDF, Bio-Rad) and incubated with the primary antibody, Phospho-Histone H2A.X (1:1000, #29380-1-AP, Proteintech, Rosemont, IL, USA), followed by incubation with a secondary anti-rabbit antibody (1:3000, ab7090, Abcam). HRP-conjugated anti-GAPDH antibody (1:10,000, ab9482, Abcam) was used as a loading control. The bands were developed using an enhanced chemiluminescence system (#1705060; Clarity Western ECL Substrate, BioRad) and visualized using ChemiDoc (BioRad).

### 2.7. ROS Measurement

The ROS measurements were performed using a CM-H2DCFDA probe (#C6827; General Oxidative Stress Indicator, Thermo Fisher Scientific) according to the manufacturer’s instructions. Briefly, the cells were harvested 4 h after the treatment, washed twice with PBS and incubated with a 5 μM probe for 45 min at 37 °C followed by removal of the probe solution. The cells were resuspended in PBS and transferred to the black 96-well plates. The fluorescence levels were measured using a Spark 10 plate reader (exc: 485nm/ em: 535 nm).

### 2.8. DNA Fragmentation

On the fifth day of treatment, cells were harvested, washed twice with PBS, and DNA and RNA were isolated using the AllPrep DNA/RNA Mini Kit (#80004; Qiagen, Venlo, The Netherlands). DNA and RNA concentrations were determined using a NanoDrop One spectrophotometer (Thermo Scientific). DNA was then diluted to 4 ng/µL and analyzed using the Agilent DNF-464b HS Large Fragment Kit (#51916568) on the Fragment Analyzer (both from Agilent, Santa Clara, CA, USA), following the manufacturer’s instructions. The average DNA fragment length was obtained from smear analysis using PROSize software (version 3.0.1.6).

### 2.9. QPCR

RNA isolated from cells and fresh-frozen tumor samples was converted to cDNA using the Maxima First Strand cDNA Synthesis Kit for RT-qPCR (#K1642; Thermo Fisher Scientific). Ten ng/µL of cDNA, equivalent to the RNA amount, was mixed with Power SYBR Green PCR Master Mix (#4368577; Thermo Fisher Scientific) and 0.5 μM of each primer in a total volume of 10 µL, then amplified on a QuantStudio 7 Flex (Thermo Fisher Scientific). Primer sequences are listed in Table 1. GUSB was used as the reference gene, and relative expression was calculated using the 2^−ΔΔCT^ method [20]. All samples were run in duplicate.

### 2.10. ADMET-AI Analysis

ADMET-AI tool (https://admet.ai.greenstonebio.com/) was used to predict pharmacokinetics and toxicity of the R162 inhibitor. ADMET properties were predicted from the chemical structure of input compound using the ADMET-AI platform, which applies machine learning models trained on curated datasets of bioactivity and pharmacokinetic data [21]. Model development was performed using a reference set of 2579 FDA-approved drugs obtained from the DrugBank (v5.1.10). In addition to raw ADMET predictions, the platform reports percentile ranks relative to the distribution of approved drugs, enabling contextual interpretation of predicted properties within a clinically relevant chemical space. The ADMET-AI report also included a ClinTox prediction, which estimates the potential clinical toxicity of a compound based on comparisons with drugs that either failed clinical trials because of toxicity or successfully completed clinical trials.

### 2.11. Statistical Analysis

Student’s *t*-test or Mann–Whitney test was used to compare the means or medians of two groups, depending on the distribution of the data. ANOVA was used to compare multiple groups when the data followed a normal distribution, while the Kruskal–Wallis test was applied for non-normally distributed data, with a Bonferroni correction for multiple comparisons. In the TCGA cohort, the optimal cut-off for dichotomizing *GLUD1* expression in Kaplan–Meier survival analyses was determined using maximally selected rank statistics implemented in the maxstat R package, which identifies the threshold that best discriminates patients according to overall survival. A single threshold of 12.61 was identified and applied consistently across all glioma subtypes. To assess whether the observed associations were dependent on cut-off-based dichotomization, *GLUD1* expression was additionally analyzed as a continuous variable after z-score transformation in Cox proportional hazards models. In multivariable analyses, models for GBM *IDH*-wildtype were adjusted for patient age, sex, and *O^6^methylguanine DNA-methyltransferase (MGMT)* promoter methylation status, whereas models for oligodendroglioma and astrocytoma were adjusted for patient age and tumor grade. Because of limited availability of clinical data in the TCGA cohort, additional potentially relevant clinical covariates could not be included. The Kaplan–Meier method with log-rank test was also used to evaluate statistical differences in survival between distinct molecular clusters. Survival analyses were performed using the survival and survminer packages in RStudio. A *p*-value below 0.05 was considered statistically significant. Each experiment was performed in triplicate for cell culture. Statistical analyses were conducted using GraphPad Prism 10.4.1 or R Studio (version 4.2.0).

## 3. Results

### 3.1. GLUD1 Expression Correlates with Molecular Classification and Survival in Gliomas

**Figure 2:**
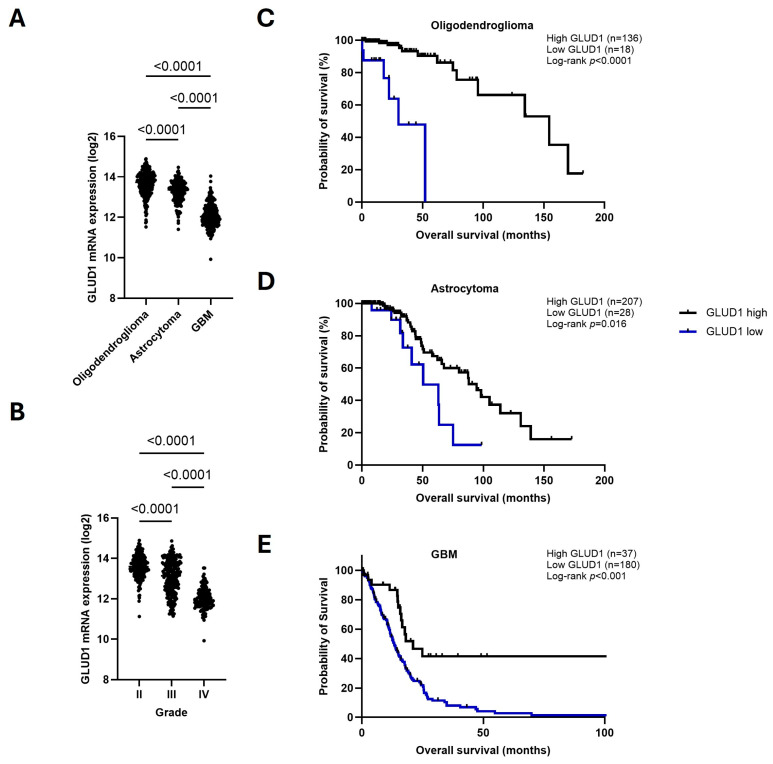
GLUD1 expression is associated with clinicopathological features and survival in gliomas based on TCGA data. (**A**,**B**) GLUD1 expression levels in glioma patients stratified by molecular subtype (**A**) and tumor grade (**B**). (**C**–**E**) Kaplan–Meier curves showing overall survival in oligodendroglioma (**C**), astrocytoma (**D**) and GBM (**E**) according to GLUD1 expression. Patients were categorized into high and low GLUD1 expression groups using cutoffs determined by maximally selected rank statistics. Survival differences were assessed using the log-rank test. *p*-values were determined using one-way ANOVA test (**A**,**B**).

To investigate the clinical relevance of GLUD1, we analyzed its expression in relation to clinicopathological features in The Cancer Genome Atlas (TCGA) glioma cohort. *GLUD1* expression differed significantly across molecular subtypes (Figure 2A,B), with higher expression observed in *IDH*-mutant gliomas compared to GBM *IDH*-wildtype (oligodendroglioma vs. GBM *IDH*-wildtype and astrocytoma vs. GBM *IDH*-wildtype, both *p* < 0.0001; Figure 2A). Although the differences between *IDH*-mutant subtypes were modest, oligodendroglioma exhibited significantly higher *GLUD1* expression than astrocytoma (*p* < 0.0001). In addition, *GLUD1* expression showed a clear association with tumor grade, with the highest levels in grade 2 and the lowest in grade 4 gliomas.

We next assessed the relationship between *GLUD1* expression and overall survival (OS). Kaplan–Meier analysis using an optimal cut-off (see Methods) demonstrated that higher *GLUD1* expression was consistently associated with improved OS in astrocytoma (94.5 vs. 50.5 months, *p* = 0.016), oligodendroglioma (154.4 vs. 30.2 months, *p* < 0.0001), and GBM *IDH* wildtype (21.0 vs. 13.3 months, *p* < 0.001; Figure 2C–E). Consistent results were obtained in univariate Cox proportional hazards models treating *GLUD1* expression as a continuous variable (Table 2), supporting the robustness of the association between *GLUD1* expression and OS across the three glioma subtypes. In multivariable analyses, the association between *GLUD1* expression and OS remained statistically significant in GBM *IDH*-wildtype, whereas it was attenuated after adjustment for age and tumor grade in astrocytoma and oligodendroglioma (Table 2). Collectively, these findings demonstrate a consistent univariate association between higher *GLUD1* expression and improved survival across glioma subtypes, while the independence of this association from established clinical prognostic factors appears to vary according to molecular subtype.

### 3.2. GLUD1 Expression Provides More Consistent Prognostic Stratification Across Glioma Sub-Types than Broader Glutaminolysis-Related Transcriptional Signatures

To assess whether the prognostic associations observed for *GLUD1* were also reflected in broader glutaminolysis-related transcriptional programs, we conducted unsupervised hierarchical clustering of TCGA glioma samples based on the expression profiles of glutaminolysis-related genes (GRGs; *GLUD1*, *GOT1/2*, *GLS*, *GLS2*, *SLC1A5*, *SLC1A7*, *GLUL,* and *GPT1/2*). By jointly analyzing *IDH* mutant and GBM *IDH*-wildtype tumors, we further assessed whether GRG expression patterns are influenced by *IDH*-mutation status. This analysis resolved gliomas into three transcriptionally distinct clusters defined by differential GRG expression (Figure S1A). Cluster 1 was characterized by uniformly low GRG expression and encompassed the majority of GBM *IDH*-wildtype tumors (88%) and a minority of *IDH* mutant tumors (19%). The remaining GBM *IDH*-wildtype and *IDH*-mutant tumors were distributed across clusters 2 and 3 without clear segregation according to *IDH*-mutation status. Likewise, oligodendrogliomas and astrocytomas were present across the same clusters, suggesting that GRG expression patterns are influenced by factors beyond *IDH* status and histological subtype. Survival analysis within glioma subtypes revealed that cluster membership was significantly associated with clinical outcome in oligodendrogliomas and GBM *IDH*-wildtype, but not in astrocytomas (Figure S1B). Notably, patients in Cluster 1, exhibiting the lowest GRGs expression, including *GLUD1*, consistently displayed the poorest prognosis in both oligodendrogliomas and GBM *IDH*-wildtype. Together, these results demonstrate that clusters defined by GRG expression differ in clinical outcome in oligodendrogliomas and GBM *IDH*-wildtype, with low *GLUD1* expression emerging as a consistent feature of the poorest-prognosis cluster.

### 3.3. GLUD1 Inhibitor R162 Effectively Reduces Glioma Cell Viability

**Figure 3:**
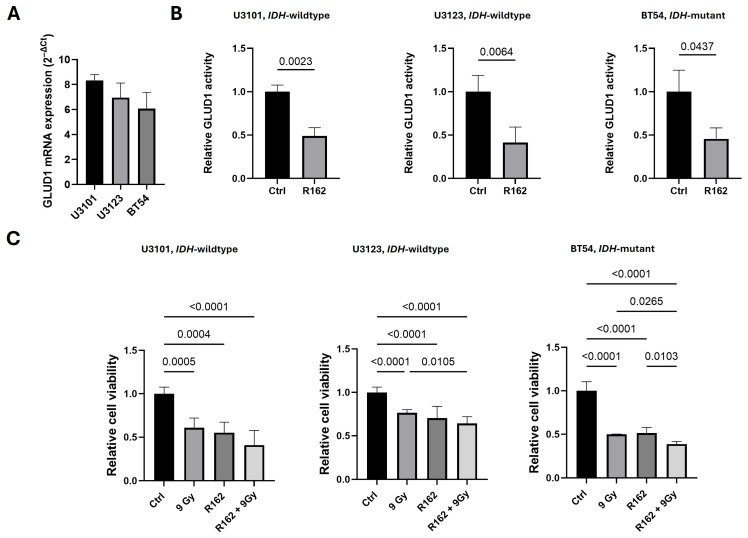
R162-mediated inhibition of GLUD1 reduces glioma cell viability. (**A**) Baseline GLUD1 expression was measured by qPCR in GBM U3101 and U3123 cell lines, and in the IDH mutant oligodendroglioma BT54 cell line (mRNA expression was normalized to internal control GUSB mRNA in all the experiments). (**B**) Glioma cells were treated with the R162 inhibitor (20 μM) or vehicle control (1% DMSO) for 1 h and GLUD1 activity was measured. (**C**) Glioma cells were treated with R162 (20 µM) and/or radiation (9 Gy) and cell viability was measured after 5 days. Data are presented as mean ± SD from three independent replicates. Statistical significance was determined using one-way ANOVA (**A**,**C**) and Student’s *t*-test (**B**).

In light of the importance of GLUD1 among GRGs demonstrated above, we next examined the cellular consequences of its inhibition in glioma by treating primary glioma cell lines with R162, a reported inhibitor of GLUD1 enzymatic activity. The characteristics and chemical structure of R162, together with the dose–response analyses, are presented in Figure S2. A concentration of 20 μM R162 was selected based on dose–response analyses, which indicated that this concentration was close to the estimated IC50 across the three cell lines (Figure S2). The experimental panel comprised two GBM *IDH*-wildtype cell lines (U3101, mesenchymal subtype, and U3123, classical subtype) and one *IDH*-mutant, oligodendroglioma cell line (BT54). Baseline *GLUD1* expression did not differ significantly among the analyzed cell lines (Figure 3A). Consistent with its proposed mechanism of action, R162 treatment resulted in a significant reduction in GLUD1 enzymatic activity compared to controls (Figure 3B). Cells were exposed to R162, radiation or a combination of both, and cell viability was assessed thereafter. Treatment with the GLUD1 inhibitor alone, as well as radiation alone, resulted in an approximately 50% reduction in cell viability across all cell lines, although the effect was less pronounced in U3123 cells (Figure 3C). The addition of radiation to R162 did not further decrease cell viability in any of the cell lines tested. To explore potential metabolic compensation following GLUD1 inhibition, we examined the expression of alternative enzymes capable of catalyzing the conversion of glutamate to α-KG, including aspartate aminotransferase (*GOT1/2*) or alanine aminotransferase (*GPT*). Among these, only *GOT1* expression was significantly elevated in GBM *IDH* wildtype cell lines, while no corresponding changes were observed in the BT54 cell line (Figure S3). Together, these data show that GLUD1 inhibition reduces glioma cell viability and is accompanied by an increase in *GOT1* expression in GBM *IDH*-wildtype cell lines.

### 3.4. GLUD1 Inhibition Induces Oxidative Stress and DNA Damage Accumulation Independent of Fumarate

**Figure 4:**
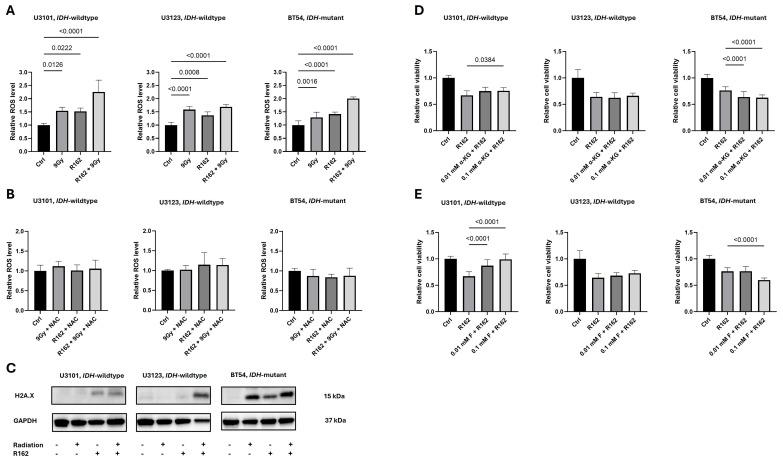
R162-mediated GLUD1 inhibition increases oxidative stress and DNA damage, and glioma cell viability is not rescued by supplementation with TCA intermediates. (**A**) Glioma cells were treated with R162 (20 µM) and/or radiation (9 Gy) and intracellular reactive oxygen species (ROS) were measured after 4 h. (**B**) ROS levels were assessed under the same treatment conditions in the presence of N-acetylcysteine (NAC, 1mM). (**C**) Glioma cells were lysed, and Western blot analysis was performed using an anti-γ-H2AX antibody, with GAPDH as a loading control. (**D**,**E**) The viability of glioma cells was measured following R162 treatment in the presence of 0.01 and 0.1 mM of dimethyl-α-ketoglutarate (DM-αKG) (**D**) or fumarate (**E**) after 5 days. Data are presented as mean ± SD from three independent replicates. Statistical significance was determined using one-way ANOVA.

Given prior evidence linking GLUD1 activity to the regulation of cellular redox balance [15,22], we examined whether pharmacological inhibition of GLUD1 with R162 induces oxidative stress in glioma cells and whether this stress contributes to downstream cellular damage. Cells were treated with R162, radiation or their combination, and intracellular reactive oxygen species (ROS) levels were assessed. As illustrated in Figure 4A, treatment with R162 alone induced ROS levels comparable to radiation alone, while the combination of both treatments resulted in a further increase in oxidative stress, the effect of which was abolished by treatment with the antioxidant N-acetylcysteine (NAC) treatment (Figure 4B). To determine whether elevated oxidative stress was associated with genomic damage, we next evaluated DNA integrity by measuring DNA fragmentation and γ-H2AX accumulation. Combined treatment with R162 and radiation increased DNA damage across all examined cell lines relative to monotherapy (Figure 4C and Figure S4). In contrast to GBM *IDH* wildtype cell lines, BT54 cells exhibited elevated γ-H2AX levels after either treatment alone, indicating increased baseline sensitivity (Figure 4C). Finally, to assess whether these effects were linked to depletion of TCA cycle intermediates previously implicated in GLUD1-mediated redox regulation [22], cells were co-treated with cell-permeable dimethyl-α-KG (DM-α-KG) or dimethyl-fumarate in the presence of R162. Supplementation with either metabolite did not restore cell viability following GLUD1 inhibition in any of the tested glioma cell lines (Figure 4D,E). These findings suggest that R162-induced oxidative stress and DNA damage are unlikely to be primarily driven by depletion of TCA cycle intermediates.

### 3.5. R162 May Cause Cellular Damage Through Glutamate and Calcium Imbalance

**Figure 5:**
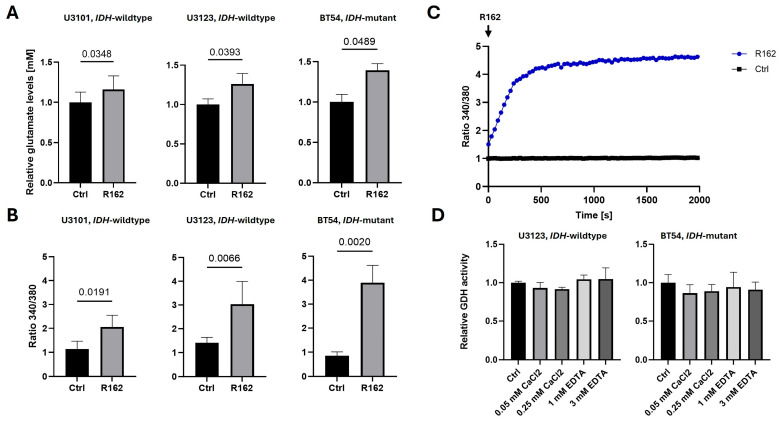
Effects of GLUD1 inhibition on glutamate and calcium homeostasis in glioma cells. (**A**) Glioma cells were treated with R162 (20 µM) or vehicle control (1% DMSO) for 24 h and intracellular glutamate levels were measured. (**B**,**C**) Glioma cells were loaded with the calcium indicator Fura-2, and intracellular calcium levels were assessed by measuring the fluorescence ratio at 340/380 nm. (**D**) The cell lysates were incubated with 0.05 or 0.25 mM of CaCl_2_ or with 1 or 3 mM of EDTA for 10 min and GLUD1 activity was measured. Data are presented as mean ± SD from three independent replicates. Statistical significance was determined using Student’s *t*-test.

We observed that pharmacological inhibition of GLUD1 with R162 altered redox homeostasis in both GBM *IDH*-wildtype and *IDH*-mutant oligodendroglioma cell lines; however, this effect appeared independent of the previously described GLUD1-fumarate-glutathione peroxidase axis. Given the established role of GLUD1 in regulating glutamate levels, we next investigated whether GLUD1 inhibition affected glutamate homeostasis. Treatment with R162 resulted in a small but still significant accumulation of intracellular glutamate in all the investigated glioma cell lines (Figure 5A). In parallel, we assessed intracellular calcium dynamics. Fura-2-loaded cells exhibited a rapid increase in intracellular free calcium ions following R162 administration in both GBM *IDH*-wildtype and *IDH*-mutant oligodendroglioma cell lines (Figure 5B,C). To determine whether calcium directly modulates GLUD1 activity, as reported for other mitochondrial dehydrogenases [23,24], we measured GLUD1 activity in the presence and absence of calcium. No differences in GLUD1 activity were detected in either condition (Figure 5D), indicating that the observed calcium dysregulation is unlikely to result from direct calcium-dependent modulation of GLUD1 activity. As dysregulated calcium signaling is a known contributor to mitochondrial dysfunction, we applied the ADMET-AI (see Methods) in silico prediction model to evaluate whether R162 is predicted to perturb mitochondrial membrane potential. The model predicted a high likelihood of mitochondrial membrane potential disruption (score 0.93 on a 0–1 scale), placing R162 in the 99.3rd percentile of compounds in the DrugBank reference set (Table S1). Collectively, our findings indicate that R162 induces ROS-associated cellular stress accompanied by perturbations in glutamate and calcium homeostasis and is predicted to disrupt the mitochondrial membrane potential.

## 4. Discussion

In this study, we sought to clarify the role of GLUD1 in glioma biology by examining both its prognostic relevance and the cellular consequences of its inhibition. We found that high *GLUD1* expression was associated with improved patient survival in the TCGA cohort, while pharmacological inhibition of GLUD1 with R162 reduced cell viability in both an *IDH*-mutant oligo- and *IDH*-wildtype GBM cell lines. These findings suggest that GLUD1 is an important regulator of glioma cell metabolism and viability, with potential implications for both prognosis and therapeutic targeting.

Previous analyses of TCGA glioma datasets have linked *GLUD1* expression to *IDH* mutation status [10,13,15]. Consistently, in our analysis, *GLUD1* expression was higher in *IDH*-mutant than in *IDH*-wildtype gliomas and was also associated with tumor grade, showing the highest levels in grade 2 and the lowest levels in grade 4 tumors (Figure 2). Higher *GLUD1* expression was associated with improved survival across glioma subtypes (Figure 2). This association remained statistically significant after adjustment for available clinical parameters in GBM *IDH*-wildtype, supporting a potential independent prognostic role of GLUD1 in this subtype (Table 2). In astrocytoma and oligodendroglioma, the association between *GLUD1* expression and survival was attenuated and did not remain statistically significant after adjustment for patient age and tumor grade. However, the relatively low number of events in these subgroups (44 and 19 events, respectively) limits the precision and statistical power of the multivariable analyses, and further studies are needed to clarify the independent prognostic relevance of GLUD1 in these tumor types. Moreover, the absence of key clinical variables, e.g., treatment information, limits the interpretation of the analyses and raises the possibility of residual confounding, particularly in GBM. Importantly, the prognostic information captured by GLUD1 was not fully recapitulated by broader GRG expression patterns (Figure S1), suggesting that GLUD1 may provide information beyond the overall transcriptional activity of glutaminolysis-related enzymes in the pathway. Our findings extend previous observations by Deng et al., who reported that lower *GLUD1* expression was associated with poorer outcomes in *IDH*-mutant gliomas [13] but did not evaluate *IDH*-mutant glioma subtypes separately or include GBM *IDH*-wildtype patients. The potential link between GLUD1, glutamate metabolism, and redox homeostasis may provide a mechanistic context for these observations. Moreira Franco et al. reported that reduced *GLUD1* expression in mesenchymal GBM *IDH*-wildtype was associated with increased glutamate availability and enhanced GSH synthesis, potentially increasing antioxidant capacity and tumor cell fitness [10]. In contrast, in lower-grade, particularly *IDH1*-mutant astrocytomas, higher *GLUD1* expression was associated with reduced expression of genes involved in GSH synthesis and a phenotype potentially more susceptible to oxidative stress [10]. These observations are broadly compatible with our finding that higher *GLUD1* expression was associated with improved survival across both *IDH*-mutant and *IDH*-wildtype gliomas. Consistent with the potential importance of GSH metabolism in glioma progression, a separate study by Deng et al. reported that increased activity of a multigene GSH-metabolism signature was associated with poorer prognosis in low-grade glioma [14]. Together, these findings support a potential relationship between *GLUD1* expression, glutamate and GSH metabolism, and glioma outcome, while the precise metabolic mechanisms underlying these associations remain to be established. Our findings should also be considered in relation to the study by Zhang et al., who reported that higher *GLUD1* expression was associated with poorer outcomes in glioma, in contrast to the favorable prognostic association observed in our molecularly defined glioma subtypes [15]. At the same time, their functional studies demonstrated that genetic or pharmacological GLUD1 inhibition, including treatment with R162, suppressed glioma-cell growth in established GBM cell-line models [15]. Thus, the functional findings of Zhang et al. are consistent with our observation that GLUD1 inhibition reduces glioma-cell viability, whereas the opposite direction of the prognostic association may reflect differences in cohort composition and molecular classification. Their study was performed using a heterogeneous glioma cohort classified according to earlier WHO criteria, whereas our analyses evaluated molecularly defined glioma subtypes separately. These findings further suggest that the prognostic association of *GLUD1* expression and the functional dependence of glioma cells on GLUD1 activity may represent related but distinct biological features.

GLUD1 inhibition with R162 reduced cell viability in all investigated glioma models, with the strongest effects observed in the *IDH*-mutant oligodendroglioma and mesenchymal GBM *IDH*-wildtype cell lines (Figure 3). Analysis of potential compensatory metabolic responses revealed increased *GOT1* expression in both GBM *IDH*-wildtype models following GLUD1 inhibition, whereas no such upregulation was observed in the *IDH*-mutant oligodendroglioma cells (Figure S3). In the mesenchymal GBM model, this compensatory increase in *GOT1* expression appeared insufficient to preserve viability, consistent with previous studies showing that mesenchymal GBM cells exhibit increased dependence on glutamine metabolism and enhanced sensitivity to glutaminolysis inhibition [25]. In contrast, the increased sensitivity of the *IDH*-mutant oligodendroglioma cell line may result from reduced metabolic adaptability associated with altered α-KG homeostasis. Although the oncometabolite 2-HG has been reported to directly inhibit GOT1 activity [26], our findings rather suggest impaired adaptive regulation of *GOT1* expression. Because α-KG functions as a critical cofactor for α-KG-dependent dioxygenases involved in epigenetic and transcriptional regulation, reduced α-KG availability following GLUD1 inhibition may impair the compensatory upregulation of metabolic enzymes such as GOT1, potentially contributing to the increased sensitivity of *IDH*-mutant cells to GLUD1 inhibition. Further studies are required to clarify whether α-KG-dependent epigenetic regulation underlies the impaired adaptive induction of GOT1 in *IDH*-mutant tumors.

Mechanistically, the reduction in glioma cell viability following pharmacological inhibition of GLUD1 was associated with disruption of intracellular glutamate homeostasis (Figure 5). Elevated glutamate levels were accompanied by increased ROS levels (Figure 4), indicating oxidative stress as a key downstream mediator, and followed by enhanced DNA damage (Figure 4 and Figure S3). Radiation—an independent inducer of ROS—did not further reduce cell viability beyond GLUD1 inhibition alone (Figure 3). However, it significantly amplified ROS accumulation and γ-H2AX formation compared to either treatment alone (Figure 4), suggesting induction of sublethal stress responses that does not translate into additive cytotoxicity. Glutamate accumulation was also accompanied by increased intracellular calcium levels, as demonstrated by Fura-2-based calcium measurements (Figure 5). Because GLUD1 enzymatic activity itself was not calcium-dependent (Figure 5), these findings suggest that calcium dysregulation occurs downstream of GLUD1 inhibition rather than serving as a regulator of enzyme activity. Given the established role of intracellular calcium imbalance in promoting oxidative stress, further studies will be required to determine how altered calcium signaling contributes to the cytotoxic effects of GLUD1 inhibition in glioma cells.

Previous studies have linked GLUD1-mediated redox regulation to modulation of the TCA cycle intermediates [22]. Jin et al. demonstrated that GLUD1 inhibition reduces α-KG and fumarate availability, thereby impairing glutathione peroxidase activity and elevating ROS [22]. However, in our study, supplementation with either α-KG or fumarate failed to rescue cell viability following R162 treatment (Figure 4), indicating that GLUD1 dependency in glioma cells is not primarily driven by impaired anaplerosis. This interpretation is consistent with previous reports showing that glutaminolysis inhibition in GBM stem-like cells is not rescued by α-KG supplementation [27]. Furthermore, fumarate-dependent redox regulation varies substantially across tumor types; for example, in fumarate hydratase-deficient renal carcinomas, fumarate accumulation directly impairs GSH function rather than enhancing antioxidant capacity [28]. Together, these observations suggest that, in glioma cells, GLUD1 inhibition induces oxidative stress through mechanisms distinct from the canonical fumarate–glutathione peroxidase axis. Computational ADMET-AI modeling further predicted a potential perturbation of mitochondrial membrane potential by R162. Although this prediction requires experimental validation, it raises the possibility that mitochondrial dysfunction may contribute to the cellular effects observed following R162 treatment.

Our findings suggest that GLUD1 may have both prognostic and therapeutic relevance in glioma. Higher *GLUD1* expression was associated with improved survival across glioma subtypes, supporting its potential as a prognostic biomarker, although validation in independent cohorts is warranted. Given that *GLUD1* expression can be readily assessed by qPCR, its incorporation into routine molecular profiling may be feasible. At the same time, pharmacological inhibition of GLUD1 reduced glioma cell viability, indicating that GLUD1-mediated metabolism may represent a potential therapeutic vulnerability. While these observations may appear contradictory, they likely reflect the distinction between prognostic association and functional dependency. Baseline *GLUD1* expression may primarily reflect the biological and metabolic state of the tumor rather than its dependence on GLUD1 activity. The higher *GLUD1* expression observed in lower-grade gliomas in our cohort is consistent with the possibility that *GLUD1* expression reflects features of tumor biology associated with a less aggressive disease state. However, this association does not imply that glioma cells are independent of GLUD1 activity. Acute pharmacological inhibition of GLUD1 instead disrupted metabolic homeostasis, leading to glutamate accumulation, oxidative stress, and reduced cell viability. The association of higher *GLUD1* expression with favorable patient outcome therefore does not preclude GLUD1 from representing a therapeutically exploitable metabolic vulnerability. Future studies should determine whether specific molecular or metabolic characteristics, rather than *GLUD1* expression alone, predict sensitivity to GLUD1 inhibition.

Several limitations should be considered when interpreting the pharmacological experiments. First, the experimental panel did not include an astrocytoma cell line, and therefore the effects of GLUD1 inhibition could not be assessed across all major glioma subtypes. This limitation reflects, in part, the difficulty of establishing and maintaining IDH-mutant primary glioma cell lines [29]. Second, our functional experiments relied on a single pharmacological GLUD1 inhibitor, R162. Previous studies have identified GLUD1 as a possible molecular target of R162 and provided evidence supporting its inhibitory activity toward GLUD1 [15,22]. In the present study, R162 was used primarily as a pharmacological tool to investigate the cellular consequences of GLUD1 inhibition, and potential off-target effects were not independently assessed. Therefore, although the observed phenotypic changes are consistent with the reported inhibition of GLUD1, we cannot exclude the possibility that some effects may have been influenced by additional molecular targets of R162. Complementary genetic or pharmacological approaches will therefore be important to further validate the specificity of the observed cellular effects. Finally, although our in silico analysis predicted a low probability of clinical toxicity for R162 compared with compounds associated with toxicity-related clinical trial failure, such predictions cannot replace experimental assessment of toxicity in non-neoplastic human brain cells.

## 5. Conclusions

We define the glioma subtype-specific prognostic relevance of GLUD1 and characterize the cellular consequences of its pharmacological inhibition, revealing a glutamate-associated oxidative stress and DNA damage response. Together, these findings distinguish *GLUD1* expression as a prognostic feature from GLUD1 activity as a potential metabolic vulnerability across glioma subtypes.

## Figures and Tables

**Table 1:** Primer sequences used in this study.

Gene	Forward Primer	Reverse Primer
GPT2	GGCTTTGGGCAGAGGGAA	TCACGCGTACTTCTCCAGGAA
GOT1	AGCTGTGCTTCTCGTCTTGC	AGATTGCACACCTCCTACCC
GOT2	GACCAAATTGGCATGTTCTGT	CGGCCATCTTTTGTCATGTA
GUSB	CAAGACAGTGGGCTGGTGAATTA	CTTGAACAGGTTACTGCCCTTGAC

**Table 2:** Univariate and multivariate Cox regression analysis in adult-type diffuse gliomas.

Subtype	Characteristics	Univariate ^1^			Multivariate ^1^		
		HR	95% CI	*p*-Value	HR	95% CI	*p*-Value
Astrocytoma (n = 235)	GLUD1 expression	0.58	0.37–0.93	0.02	0.6	0.34–1.04	0.07
	Age	1.02	0.99–1.05	0.14	1.02	0.99–1.05	0.16
	Tumor grade: 3	1.51	0.82–2.81	0.19	1.52	0.82–2.85	0.19
	4	3.58	1.21–10.63	0.02	2.9	0.95–9.1	0.08
Oligodendroglioma (n = 154)	GLUD1 expression	0.24	0.11–0.53	0.0004	0.87	0.32–2.3	0.78
	Age	1.13	1.07–1.19	<0.0001	1.12	1.05–1.19	0.0005
	Tumor grade: 3	6.76	2.12–21.53	0.001	4.9	1.4–17.3	0.01
GBM (n = 217)	GLUD1 expression	0.61	0.46–0.8	0.0003	0.66	0.48–0.9	0.0085
	Patient age	1.03	1.02-1.05	<0.0001	1.03	1.02–1.05	<0.0001
	Sex: Male	1.2	0.88–1.7	0.21	0.97	0.67-1.4	0.86
	MGMT promoter: Unmethylated	0.9	0.63–1.3	0.59	1.16	0.79–1.7	0.44

^1^ In both univariate and multivariate Cox regression analyses, GLUD1 expression and patient age were evaluated as continuous variables, whereas tumor grade, sex, and MGMT promoter methylation status were evaluated as categorical variables. *p* < 0.05 is shown in bold. MGMT, methylguanine-DNA methyltransferase.

## Data Availability

The publicly available TCGA GBMLGG dataset analyzed in this study can be accessed through the Gliovis platform (https://gliovis.bioinfo.cnio.es/, accessed on 20 May 2026). The original data generated and analyzed during this study are included in this published article and its Supplementary Materials. Further information and materials are available from the corresponding author upon reasonable request.

## Supplementary Materials

The following supporting information can be downloaded at: https://www.mdpi.com/article/10.3390/cells15151401/s1, Figure S1: Expression-based clustering of glutaminolysis-related genes in glioma patients and associated overall survival. (A) TCGA-GBMLGG glioma samples were grouped by unsupervised hierarchical clustering based on the differential expression of glutaminolysis-related genes (GRGs). Heatmap colors represent relative gene expression levels. (B–D) Kaplan–Meier curves showing overall survival of patients with oligodendroglioma (B), astrocytoma (C), and GBM (D) according to cluster membership. Figure S2: R162 inhibitor characterization and dose–response analysis. (A) Chemical structure and molecular weight of R162. (B–D) Glioma cell lines were treated with different concentrations of reported GLUD1 inhibitor R162 molecule and cell viability was evaluated after 5 days. Dose–response curve in BT54 (B), U3101 (C) and U3123 (D) cell lines. Figure S3: In IDH-wildtype gliomas, the inhibition of GLUD1 increases GOT1 expression. Glioma cells were treated with R162 (20 μM) or vehicle control (1% DMSO) for 5 days and expression of GOT1 was quantified by qPCR. Data are presented as mean ± SD from three independent replicates. Statistical significance was determined using Student’s *t*-test. Figure S4: Treatment with either R162 inhibitor or radiation induces DNA damage, which is further enhanced under combined treatment. Glioma cells were treated with R162 (20 μM) and/or radiation (9 Gy) and DNA fragmentation was quantified by capillary electrophoresis. Data are presented as mean ± SD from three independent replicates. Statistical significance was determined using one-way ANOVA. Table S1: ADMET-AI prediction profile of R162. Selected DrugBank percentile rankings generated using ADMET-AI.

## Abbreviations

The following abbreviations are used in this manuscript:

A-KG	α-ketoglutarate
TCGA	The Cancer Genome Atlas
TCA	tricarboxylic acid
SLC1A5	solute carrier family 1 member 5
ROS	reactive oxygen species
NAC	N-acetylcysteine
IDH	isocitrate dehydrogenase
GUSB	beta-glucuronidase
GSH	glutathione
GRG	glutaminolysis-related gene
GPT	alanine aminotransferase
GOT	aspartate aminotransferase
GLUL	glutamine synthetase
GLUD1	glutamate dehydrogenase 1
GLS	glutaminase
GBM	glioblastoma
DM-αKG	dimethyl α-ketoglutarate
2-HG	2-hydroxyglutarate
MGMT	Methylguanine DNA-methyltransferase
