# Supplementary material for: GLUD1 Inhibition Disrupts Glutamate Homeostasis and Induces Metabolic and Redox Stress in Gliomas
Source: Cells. 2026 Aug 3;15(15):1401. doi: 10.3390/cells15151401 (PMC13465080; doi:10.3390/cells15151401)
Supplement: Supplementary file 1 [file cells-15-01401-s001.zip › cells-4442317-supplementary.pdf]

Supplementary Materials

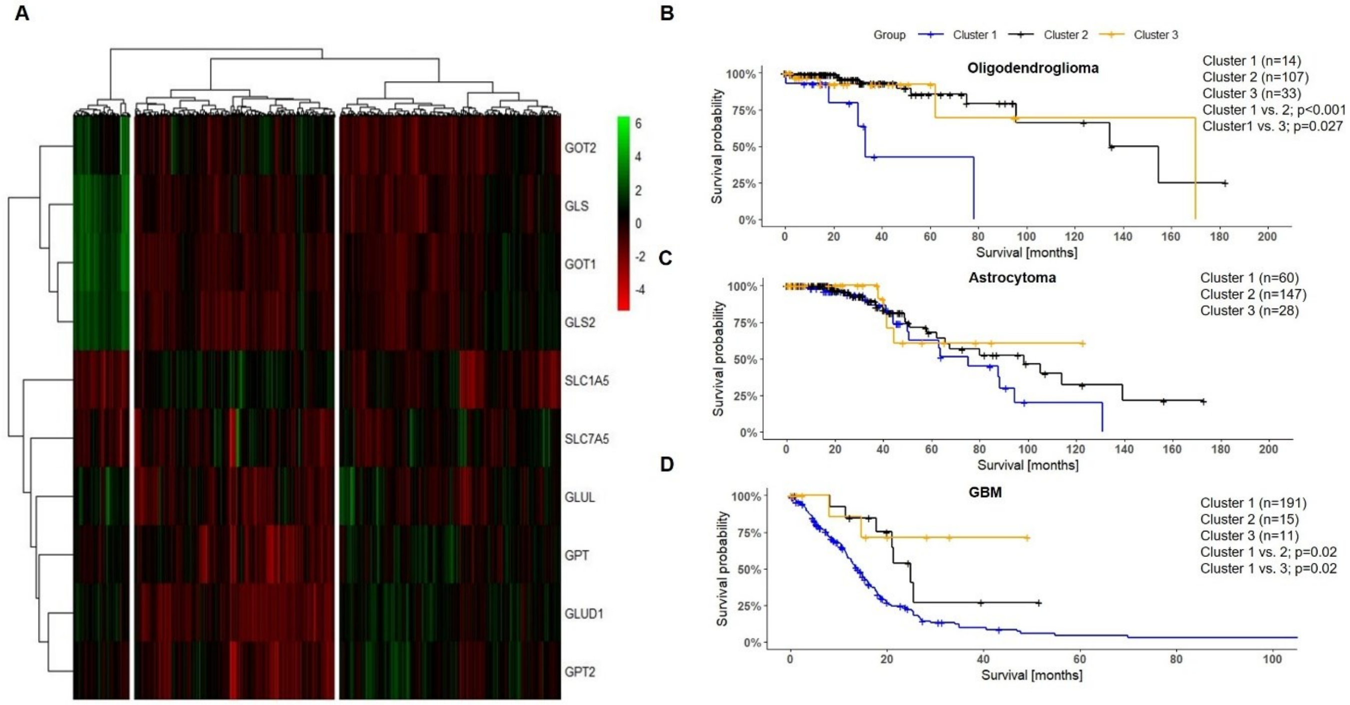

Figure S1. Expression-based clustering of glutaminolysis-related genes in glioma patients and associated overall survival. (**A**) TCGA-GBMLGG glioma samples were grouped by unsupervised hierarchical clustering based on the differential expression of glutaminolysis-related genes (GRGs). Heatmap colors represent relative gene expression levels (**B–D**) Kaplan-Meier curves showing overall survival of patients with oligodendroglioma (**B**), astrocytoma (**C**), and GBM (**D**) according to cluster membership.

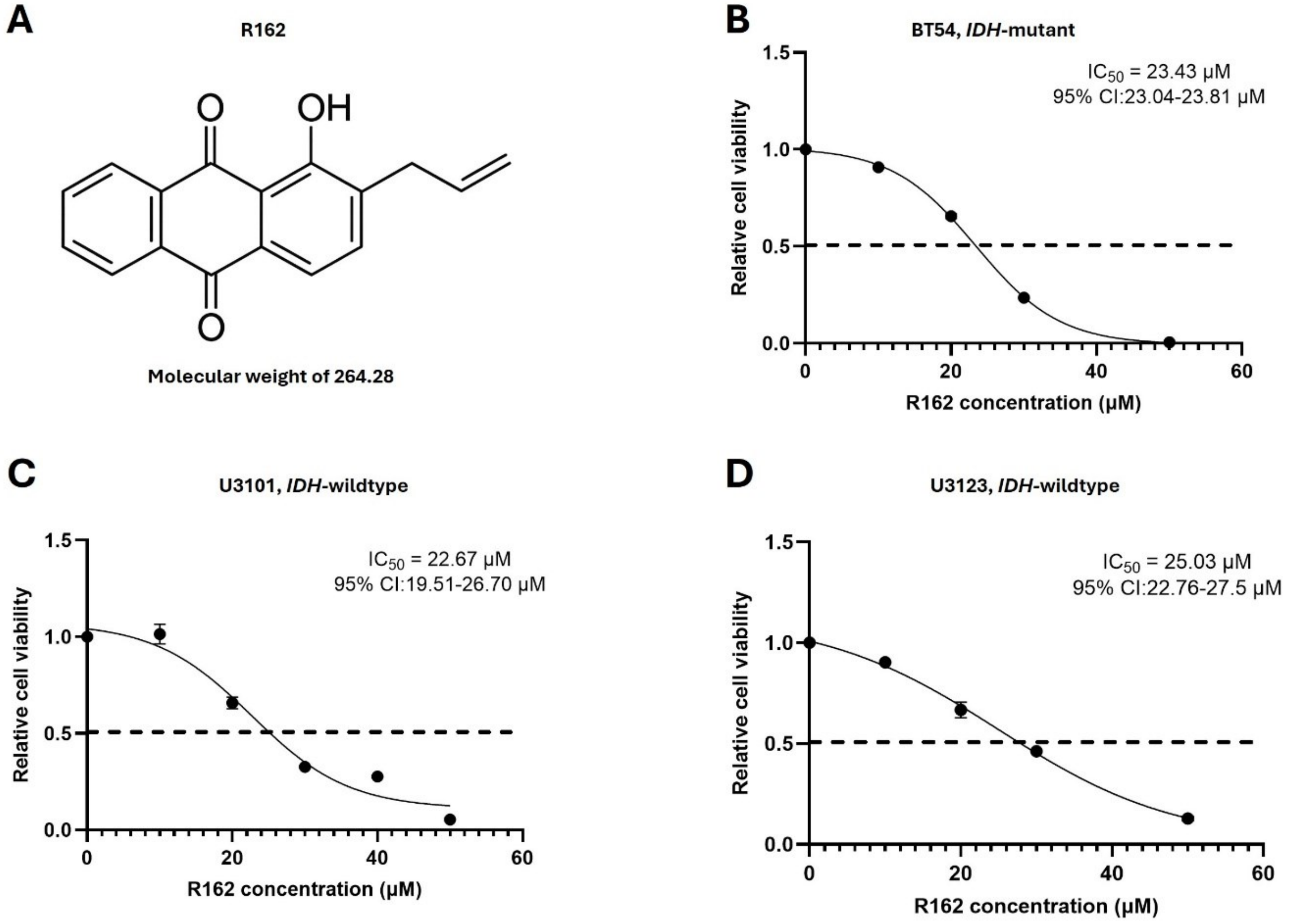

Figure S2. R162 inhibitor characterization and dose–response analysis. (**A**) Chemical structure and molecular weight of R162. (**B–D**) Glioma cell lines were treated with different concentrations of reported GLUD1 inhibitor R162 molecule and cell viability was evaluated after 5 days. Dose-response curve in BT54 (**B**), U3101 (**C**) and U3123 (**D**) cell lines.

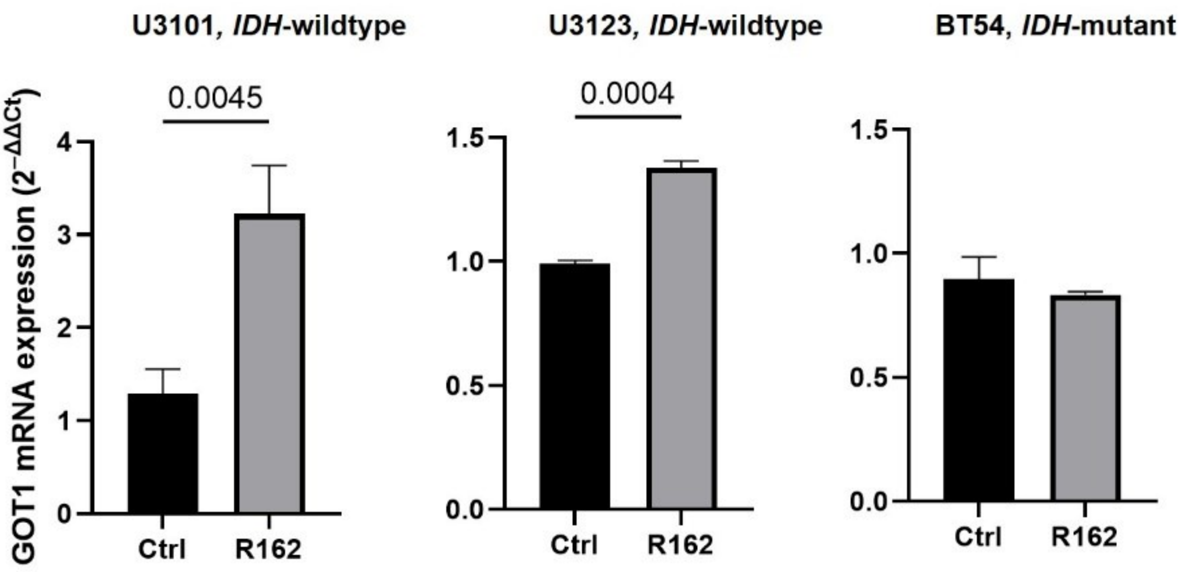

Figure S3. In IDH-wildtype gliomas, the inhibition of GLUD1 increases *GOT1* expression. Glioma cells were treated with R162 (20 μM) or vehicle control (1% DMSO) for 5 days and expression of *GOT1* was quantified by qPCR. Data are presented as mean ± SD from three independent replicates. Statistical significance was determined using Student's t-test.

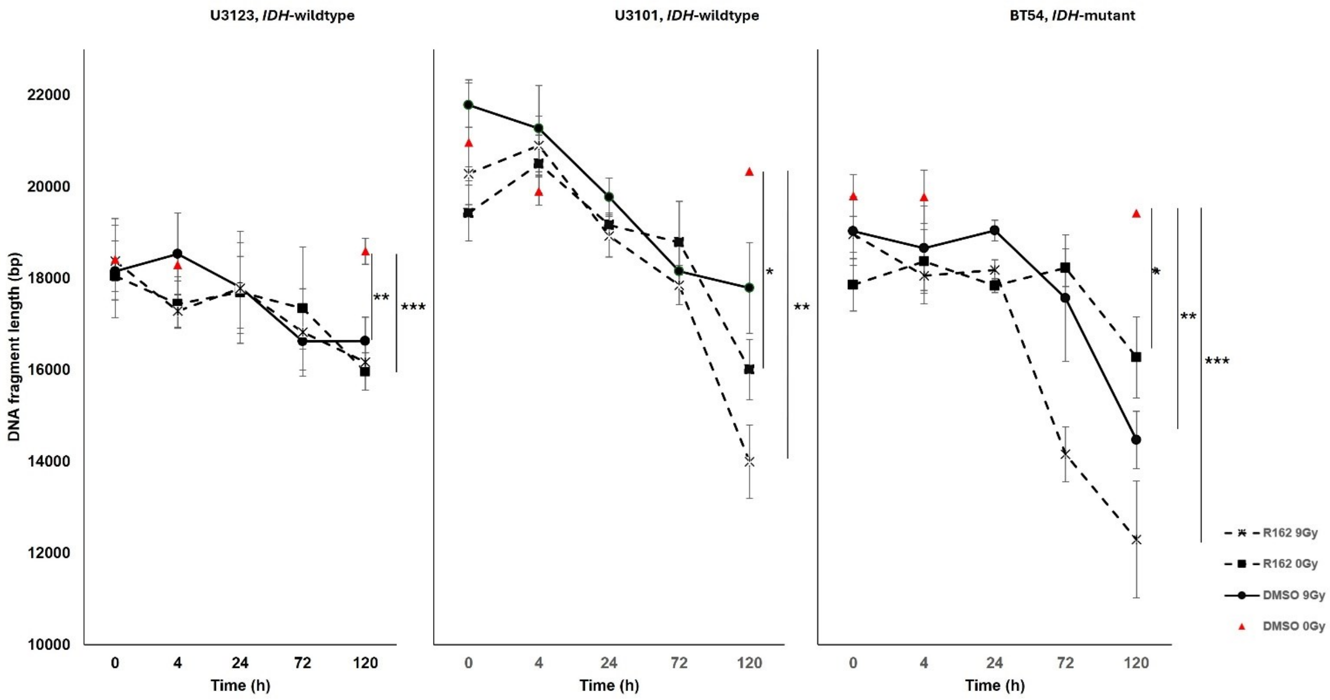

Figure S4. Treatment with either R162 inhibitor or radiation induces DNA damage, which is further enhanced under combined treatment. Glioma cells were treated with R162 (20 μM) and/or radiation (9 Gy) and DNA fragmentation was quantified by capillary electrophoresis. Data are presented as mean ± SD from three independent replicates. Statistical significance was determined using one-way ANOVA. * $p < 0.05$, ** $p < 0.01$, *** $p < 0.001$.

Table S1. ADMET-AI prediction profile of R162. Selected DrugBank percentile rankings generated using ADMET-AI.

Property	Prediction	DrugBank percentile
**Molecular weight**	264.3 Da	
**LogP**	2.9	60.3
**Oral bioavailability**	0.52	20.7
**Blood-brain barrier penetration**	0.48	29.1
**Half Life**	19 hr	66
**Drug clearance (Hepatocyte)**	62 uL/min/mg	71.7
**Clinical toxicity**	0.05	36.5
**Drug-induced liver injury**	0.9	80.9
**Skin reaction**	0.93	95.7
**Mitochondrial membrane potential disruption**	0.93	99.3
